# Supplementary material for: CoVM2: Molecular Biological Data Integration of SARS-CoV-2 Proteins in a Macro-to-Micro Method
Source: Biomolecules. 2022 Aug 2;12(8):1067. doi: 10.3390/biom12081067 (PMC9405999; doi:10.3390/biom12081067)
Supplement: Supplementary file 1 [file biomolecules-12-01067-s001.zip › prodigy_results/ACE2_soba1/result.html]

PRODIGY - Run


# PRODIGY

## @Bonvinlab


*home*

Home

- PRODIGY
- PRODIGY

  PRODIGYHADDOCK2.2
  HADDOCK2.4
  DISVIS
  CPORT
  POWERFIT
  SPOTON
  PROABC2
  WHISCY
  PDBTOOLS
- Home
- Manual
- Method
- Dataset
- Example
- Reference
- Help


Welcome to the Utrecht Biomolecular Interaction Web Portal >>

**Your PRODIGY prediction () has finished successfully.**

Please note that the results will be deleted after two weeks.

**To cite PRODIGY**, please refer to:

1. Vangone A. and Bonvin A.M.J.J. **"Contact-based prediction of binding affinity
   in protein-protein complexes"**, *eLife*, 4, e07454 (2015).
2. Xue L., Rodrigues J., Kastritis P., Bonvin A.M.J.J.\*, Vangone A.\*,**"PRODIGY: a web-server for predicting the binding affinity in protein-protein complexes"**,
   *Bioinformatics*, doi:10.1093/bioinformatics/btw514 (2016).

For more information about the **predictive model** please check the
PRODIGY prediction method page.

Further information can be found in the PRODIGY Manual
and an example of the PRODIGY output can be found here.

Back to PRODIGY home page

**How would you rate your experience with our portal?** 
*sentiment\_very\_dissatisfied*
*sentiment\_dissatisfied*
*sentiment\_neutral*
*sentiment\_satisfied*
*sentiment\_very\_satisfied*

Thank you! *done*

## Binding affinity and Kd prediction

The binding affinity (ΔG) and dissociation constant (Kd)predicted values are:

| Protein-protein complex | ΔG (kcal mol-1) | Kd (M) at 37.0 ℃ |
| --- | --- | --- |
| 7wbp | -11.1 | 1.5E-08 |

## Prediction details

**Number of Interfacial Contacts (ICs) per property:**

|  |  |
| --- | --- |
| ICs charged-charged: | 7 |
| ICs charged-polar: | 7 |
| ICs charged-apolar: | 18 |
| ICs polar-polar: | 5 |
| ICs polar-apolar: | 17 |
| ICs apolar-apolar: | 11 |

**Non Interacting Surface (NIS) per property:**

|  |  |
| --- | --- |
| NIS charged: | 25.78% |
| NIS apolar: | 35.37% |

**Table of the ICs at the interface:**

|  |
| --- |
| List of residue-residue pairs at the interface (.txt) |

## Download outputs

|  |
| --- |
| Pymol ICs-based representation script (.pml) |
| Archive file of all the outputs (.tgz) |

###### **Proudly powered by:**

This work is co-funded by the Horizon 2020 projects EOSC-hub and EGI-ACE (grant numbers 777536 and 101017567), BioExcel (grant numbers 823830 and 675728)   
and by a computing grant from NWO-ENW (project number 2019.053).

2008-2022 © Computational Structural Biology group. All rights reserved.

Webdesign by Marc van Dijk, Mikael Trellet, Jörg Schaarschmidt, Brian Jiménez-García   
& Rodrigo Vargas Honorato

Terms of use |
 Privacy
